# Assessment of clinical activity and severity using serum ANCA and ASCA antibodies in patients with ulcerative colitis

**DOI:** 10.1186/s13223-020-00433-1

**Published:** 2020-05-20

**Authors:** Yanhua Pang, Huijie Ruan, Dongfang Wu, Yanfei Lang, Ke Sun, Cuiping Xu

**Affiliations:** 1 Department of Gastroenterology, Beijing Chaoyang Hospital, Capital Medical University, Chaoyang District, Beijing, 100020 China; 2 Department of Gastroenterology, The First Hospital of Shanxi Medical University, Taiyuan, Shanxi 300001 China; 3 Department of Gastroenterology, Hebei Yanda Hospital, Hebei Medical University, Sanhe, Hebei 065201 China; 4 Department of Pathology, Shihezi University School of Medicine, Shihezi, Xinjiang 832002 China

**Keywords:** Ulcerative colitis, Anti-*Saccharomyces cerevisiae* antibody, Anti-neutrophil cytoplasmic antibody, Dynamic quantitation

## Abstract

**Background:**

Ulcerative colitis (UC) is a chronic, non-specific inflammatory bowel disease (IBD) with unknown etiology. The lack of specific clinical manifestations, standard diagnostic criteria, objective and accurate indicators to the severity of the disease and the efficacy of the treatment, often results in difficulties in diagnosis and timely treatment of UC. Therefore, there is a need to develop a clinically suitable serum biomarker assay with high specificity and sensitivity.

**Objective and methods:**

To explore the significance of anti-neutrophil cytoplasmic antibodies (ANCA) and anti-saccharomyces cerevisiae antibodies (ASCA) in the diagnosis, differential diagnosis and treatment assessment in patients with ulcerative colitis (UC). Serum levels of ANCA-IgG, ASCA-IgA and ASCA-IgG were measured by an enzyme-linked immunosorbent assay (ELISA) in 105 UC patients, 52 non-UC patients and 100 healthy controls.

**Results:**

(1) Both the ANCA-IgG level and its positive rate in UC patients were significantly higher than those in non-UC controls and healthy controls (*p* < 0.01). However, the levels of ASCA-IgA, ASCA-IgG and the positive rates in UC patients had no statistical differences when compared with those in non-UC controls or healthy controls (*p* > 0.05). (2) The sensitivity of ANCA^+^ and ANCA^+^/ASCA^−^ in detecting UC patients was 61.90% and 55.24%, respectively, whereas the specificity was 91.45% and 94.08%, respectively. The sensitivity of ASCA^+^ and ASCA^+^/ANCA^−^ in non-UC disease controls was 5.33% and 3.85%, respectively, and specificity was 83.9% and 88.78%, respectively. (3) When UC patients were grouped into mild, moderate or severe subtypes, the ANCA-IgG levels were correlated with the severity of UC, and the differences of the ANCA-IgG levels were statistically different among the three subtypes (*p* < 0.05). There was no correlation between the levels of ANCA-IgG and the disease locations of UC.

**Conclusions:**

(1) Serum levels of ANCA may be useful in the diagnosis of UC. (2) Dynamic quantitation of ANCA-IgG levels may be helpful in determining the severity of UC and therefore, may guide treatment of UC.

## Background

In theory, quantitative measurements of UC-related auto-antibodies would be helpful for the clinical diagnosis and treatment assessment of UC. Anti-neutrophil cytoplasmic antibodies (ANCA) are a group of auto-antibodies with cytoplasmic components of the neutrophils as the target antigens, which can release lysozymes through capillaries, damage blood vessels and intestine tissues, and also cause tissue damages through T cell-mediated cellular immune synergy [1, 2].

In recent years, studies have demonstrated that these ANCAs specifically mediate several diseases such as glomerulonephritis, systemic vasculitis, nodular granulomatosis and autoimmune hepatitis. An antibody named as atypical nuclear ANCA (atypical p-ANCA) has been related to UC [3, 4]. Another antibody highly associated with IBD is anti-*Saccharomyces cerevisiae* antibody (ASCA) which is directed against the yeast genus. ASCA mainly targets at peptidemimetic polysaccharide on the cell wall of the yeast. The mechanisms of these antibodies involved in IBD may be related to the increased intestinal permeability of the disease and the exposure to immune response cells of yeast antibodies [5]. By detecting serum levels of ANCA-IgG, ASCA-IgA and ASCA-IgG, we have investigated the clinical significance of these antibodies in UC diagnosis, differential diagnosis, and possible correlations of antibody levels to the disease state and to the treatment efficacy.

## Patients and methods

### Patients

Blood samples from 105 UC patients (52 males, 53 females, average age of 47.33 ± 15.43 years old) diagnosed by the outpatient and inpatient of the First Hospital of Shanxi Medical University from July 2015 to October 2016 were included in this study. The diagnosis of UC was based on clinical, endoscopic and histopathological findings in accordance with the IBD diagnostic criteria determined by the Chinese Medical Association meeting in Guangzhou [6]). Blood samples from 52 non-UC patients (28 males, 24 females, average age of 50.9 ± 14.0 years old), which were diagnosed as other intestinal disease (i.e. colitis, terminal ileitis, intestinal tuberculosis, intestinal polyps, white’s disease and crohn’s disease) according to the colonoscopyexamination, were selected as disease control. Meanwhile, 100 blood samples from healthy volunteers who came from our health examination center (54 males, 46 females, average age of 53.2 ± 14.9 years old) used in this study. There were no significant differences in both gender and average age among the three groups. According to Mayo Score System, the 105 UC serum samples were divided into three subgroups based on the disease severity: mild group (49 cases), moderate group (40 cases) and severe group (16 cases) [7]. The basic clinical data of the selected samples were shown in Table 1.

**Table 1:** Basic clinical information, serum levels and positive rates of ANCA and ASCA of the patients and controls

Patients groups	N	Male	Female	Male:Female	Average of age (yrs)	ANCA-IgG	ASCA-IgA	ASCA-IgG
						N (%)	N (%)	N (%)
Healthy controls	100	54	46	1:0.85	53.19 ± 14.85	3.15 ± 2.24	1.16 ± 1.49	3.41 ± 5.41
						6 (6)	7 (7)	8 (8)
Disease controls	52	28	24	1:0.86	50.88 ± 14.01	4.03 ± 5.96	0.95 ± 1.94	2.38 ± 3.16
						7 (13.5)	3 (5.77)	1 (1.92)
UC	105	52	53	1:1.02	47.33 ± 15.43	17.5 ± 30.6^a,b^	2.06 ± 9.18	4.05 ± 6.42
						65 (61.9)^a,b^	11 (10.5)	13 (12.4)

Age years with mean ± SD, antibody levels are U/mL (mean ± SD)

^a^*P *< 0.05: Compared with healthy controls

^b^*P *> 0.05: Compared with disease controls

### Serologic measurements

Five mL of blood samples were drawn from UC patients, disease controls and healthy controls and then centrifuged at 3000 r/min for 10 min. The serum samples were collected and stored at − 80 °C until used. Three serum antibodies (ANCA-IgG, ASCA-IgA and ASCA-IgG) were detected using ANCA-IgG, ASCA-IgA and ASCA-IgG ELISA kits manufactured by Shanxi Ruihao Biotechnology company (Shanxi, China), and all the assays were conducted according to the kit instructions. Based on the kit instruction, the cut-off values of ANCA-IgG, ASCA-IgA and ASCA-IgG were 5.03, 1.91 and 7.31 U/mL, respectively. Antibody concentration levels greater than the cut-off value were regarded as positive.

### Statistical analysis

SPSS 19.0 software package was used for statistical analysis. All the results were presented as the mean ± standard error ($\bar{x}$ ± S). The comparisons were conducted using variance analysis, and the comparison between each pair of the three groups was conducted using SNK test. The enumeration data were expressed by percentage, and the data comparison between two groups used Fisher Exact Probability test. The difference was regarded as statistically significant when *p* < 0.05.

## Results

### Serum levels of ANCA, ASCA and the positive rates among the three comparison groups

Our data suggested that: (1) The ANCA-IgG level and positive rate in UC group were significantly higher than those of disease control group and healthy control group,the differences were statistically significant (*p* < 0.01); There were no significant differences in ANCA-IgG level and positive rate between disease control group and healthy control group (*p* > 0.05); (2) There were no significant differences in ASCA-IgA, ASCA-IgG levels and positive rate among UC group, disease control group and healthy control group. Results were shown in Table 1.

### Correlations between serum autoantibody levels and the severity of UC as well as the disease locations

The level of serum ANCA-IgG in severe group was significantly higher than those in moderate group and mild group (*p *< 0.01). The serum level of ANCA-IgG was also significant higher in moderate group than that in mild group (*p *< 0.05). There were no significant differences of the serum ASCA-IgA and ASCA-IgG levels in patients among the three subgroups. Results were shown in Table 2.

**Table 2:** Serum levels of ANCA and ASCA in UC patients with different disease status

Patients groups	N	ANCA-IgG	ASCA-IgA	ASCA-IgG
UC mild	49	4.65 ± 2.81	3.36 ± 13.2	3.18 ± 5.02
UC moderate	40	14.70 ± 18.4^c^	0.83 ± 2.00	4.47 ± 7.11
UC severe	16	64.00 ± 52.1^a,b^	1.15 ± 3.04	2.86 ± 6.01

Antibody levels are U/mL (mean ± SD)

^a^Comparison between UC severe group and UC moderate group (p < 0.01)

^b^Comparison between UC severe group and UC mild group (p < 0.01)

^**c**^Comparison between UC moderate group and UC mild group (p < 0.05)

According to Montreal Classification [8], all the 105 UC serum samples were divided into three subgroups based on the lesion location such as the whole colon type group (42 cases), the left colon type group (32 cases) and the rectal type group (31 cases). The levels of serum ANCA-IgG, ASCA-IgA and ASCA-IgG had no significant differences among the three groups (*p *> 0.05). Results were shown in Table 3.

**Table 3:** Serum levels of ANCA and ASCA in UC patients with different disease locations

Disease locations	N	ANCA-IgG	ASCA-IgA	ASCA-IgG
Whole colon type	42	19.90 ± 36.00	1.45 ± 6.68	4.36 ± 6.68
Left colon type	32	20.76 ± 34.57	3.45 ± 15.90	2.90 ± 5.99
Rectal type	31	10.97 ± 13.15	1.46 ± 3.05	3.36 ± 5.12

The levels of serum ANCA and ASCA in patients within the whole colon type group, left colon type group and rectal type group were compared, and no significant difference with each other were observed (p > 0.05). Antibody levels are U/mL (mean ± SD)

### Comparative evaluation of a single ANCA test vs. combinational tests of ASCAs for UC diagnosis and differential diagnosis

Both assays’ specificity and sensitivity were used for the comparative evaluation. The sensitivity of serum ANCA^+^ and ANCA^+^/ASCA^−^ in UC group was 61.90% and 55.24%, respectively, whereas the specificity was 91.45% and 94.08%, respectively. The sensitivity of serum ASCA^+^ and ASCA^+^/ANCA^−^ in non-UC disease control group was 5.33% and 3.85%, respectively, and the specificity was 83.90% and 88.78%, respectively. The positive predictive values of the above antibodies were 83.33%, 86.57%, 8.33% and 8.00%, respectively, and the negative predictive values were 77.65%, 75.26%, 77.83% and 78.48%, respectively. These results were tabulated in Table 4.

**Table 4:** Comparative analyses of single ANCA test vs. combinational tests for ANCA/ASCA in the diagnosis of UC

Antibody/disease	Sensitivity (%)	Specificity (%)	Positive predictive value (%)	Negative predictive value (%)
ANCA^+^/UC	61.90	91.45	83.33	77.65
ANCA^+^/ASCA^−^/UC	55.24	94.08	86.57	75.26
ASCA^+^/disease control group	5.77	83.90	8.33	77.83
ASCA^+^/ANCA^−^/disease control group	3.85	88.78	8.00	78.45

More than one positive test of ASCA-IgG and ASCA-IgA subtypes was regarded positive; two negative tests of the subtypes were regarded negative

## Discussion

Ulcerative colitis (UC) is a chronic inflammatory bowel disease with onset most frequently in young adulthood. Many patients with UC have a lifetime of debilitating physical symptoms (e.g., urgent diarrhea, rectal bleeding, abdominal pain). UC is diagnosed by the combination of clinical pattern, colonoscopy, CT, MR and histological findings (basal plasmacytosis, diffuse crypt atrophy and distortion, villous surface irregularity and mucus depletion) [6]. mild-moderate fewer than 4–6 bowel movements per day, mild-moderate rectal bleeding, absence of constitutional symptoms, and absence of features suggestive of high inflammatory activity based upon Truelove and Witt’s criteria 3 and the Mayo Clinic score. Patients with mild-moderate disease activity generally are at low risk of requiring colectomy. However, certain disease features may predict an aggressive disease course. These include extensive disease, severe endoscopic activity, extra-intestinal manifestations, and elevated inflammatory markers [9]. ANCA-associated vasculitides (AAV)includes granulomatosis with polyangiitis (GPA), eosinophilic granulomatosis with polyangiitis (EGPA) and microscopic polyangiitis (MPA). Few cases of vasculitis were found associated with IBD [10].

Searching for laboratory bio-indicators related to UC diagnosis and providing a simple, objective and accurate serum marker for diagnosis, differential diagnosis and efficacy assessment are the ultimate goal in clinical gastroenterology. It has been well documented that ANCA is associated with UC. At present, the most widely used technologies for ANCA detection are indirect immunofluorescence (IIF) assay and ELISA. Since it is quite common that antinuclear antibodies (ANA) can be present at the same time when ANCA is detected with the qualitative IIF assay. It has been documented that ANCA^+^ may be missed through IIF test due to the interference of ANA, while this can be overcome when a specific ELISA method is used.

In our previous studies, we have demonstrated that quantitative ELISA assaying ANCA levels has a good consistency with IIF and yet has a sensitivity superior to IIF [11]. According to a large number of reports, ANCA is present in 40%–85% of UC patients, 5%–10% of CD patients, and 0–5% of healthy controls [12]. Bansi et al. have reported that ANCA is detected in 42.4% of UC patients but only in 5% of CD with a specificity of 98% and a sensitivity of 42%, respectively, for diagnosing UC [13]. Liu et al. have also reported that ANCA is detected in 48.6% of UC patients and in 25% of CD patients and the sensitivity of single test of ANCA for diagnosing UC is 48.6% with a specificity of 92.9% [14]. Qiu et al. have demonstrated that ANCA was detected in 68.3% of UC patients and only in 1.7% of healthy controls with a sensitivity of 65.9% and a specificity of 92.7%, respectively, in a single test of ANCA for the diagnosis of UC [15]. In this report, our results indicate that ANCA positivity is of a good clinical reference value for UC diagnosis and, the augment of ANCA levels may improve the diagnostic accuracy of UC.

At present, it is still controversial whether ANCA levels in UC patient is associated with the severity of the disease. He et al. have reported that ANCA levels are associated with the severity of the disease. Their results have shown that ANCA positive UC patients have a higher rate of intestinal mucosal vasculitis than ANCA negative UC patients. In addition, the UC patients with high ANCA levels also have a severe pathological inflammation at the intestinal mucosa [16]. However, Yang et al. have reported conflicting results [17]. In order to evaluate the correlation, UC patients have been divided into mild, moderate and severe groups based on the disease severity. Following the aggravation of the disease, levels of ANCA-IgG show upward tendency and the serum ANCA-IgG levels are indeed higher in patients in the severe group than those in the moderate and mild groups (p < 0.001). Similarly, the serum ANCA-IgG levels are also significantly higher in moderate patients than those in the mild group (p < 0.05).

A study reported that the positive rate of ASCA in CD patients was 39%–72%, higher than that of UC and disease control patients (4%–14% and 4%–14% respectively). In this study, the positive rates of ASCA -IgG and ASCA -IgA in UC patients were 13% and 11%, respectively, with no significant difference from this research data. Studies have shown that the presence of ASCA is related to small bowel involvement (especially ileum) [18], while the disease site of UC is mainly located in the colorectal, which may be one reason for the low positive rate of ASCA in UC patients. In a prospective comparative study of ASCA in IBD patients in China and whites, it was mentioned that there was a significant difference in the mutation frequency of NOD2 gene (NOD2 gene is considered to be a strongly correlated gene of CD) in CD among different ethnic groups. Currently, the sensitivity of Chinese people to ASCA detection is lower than that of white people [19].

## Conclusions

Serum ANCA^+^ levels are helpful in the diagnosis of UC, while combined tests of serum ANCA-IgG, ASCA-IgG, and ASCA-IgA levels are useful in differential diagnosis of UC. Furthermore, in UC patients, serum ANCA levels is correlated with the severity of the disease and, therefore, a dynamic monitoring of serum ANCA levels during treatment may relieve patients from repeated use of invasive diagnostic methods. The dynamic testing of serum antibody levels as mentioned above may also help monitor disease progression, guide for more effective clinical medications and reduce the recurrence of UC.

## Data Availability

The datasets used and/or analysed during the current study are available from the corresponding author on reasonable request.

## Abbreviations

ANCA: Anti-neutrophil cytoplasmic antibodies
ASCA: Anti-*Saccharomyces cerevisiae* antibodies
UC: Ulcerative colitis
IBD: Inflammatory bowel disease
ANA: Antinuclear antibodies
